# A mouse model of cochlear implantation with chronic electric stimulation

**DOI:** 10.1371/journal.pone.0215407

**Published:** 2019-04-18

**Authors:** Alexander D. Claussen, René Vielman Quevedo, Brian Mostaert, Jonathon R. Kirk, Wolfram F. Dueck, Marlan R. Hansen

**Affiliations:** 1 Department of Otolaryngology-Head and Neck Surgery, University of Iowa, Iowa City, IA, United States of America; 2 Cochlear Americas, Centennial, CO, United States of America; 3 Cochlear Deutschland GmbH & Co. KG, Hannover, Germany; 4 Department of Neurosurgery, University of Iowa, Iowa City, IA, United States of America; University of Miami School of Medicine, UNITED STATES

## Abstract

**Objectives:**

Cochlear implants provide an effective treatment option for those with severe hearing loss, including those with preserved low frequency hearing. However, certain issues can reduce implant efficacy including intracochlear tissue response and delayed loss of residual acoustic hearing. We describe a mouse model of cochlear implantation with chronic electric stimulation that can be used to study cochlear implant biology and related pathologies.

**Methods:**

Twelve normal hearing adult CBA/J mice underwent unilateral cochlear implantation and were evenly divided into one group receiving electric stimulation and one not. Serial impedance and neural response telemetry (NRT) measurements were made to assess implant functionality. Functionality was defined as having at least one electrode with an impedance ≤ 35 kOhms. Mouse cochleae were harvested for histology and 3D x-ray microscopy 21 days post-operatively, or, in case the implant was still functional, at a later time point when the implant failed. A separate experiment measured the hearing preservation rate in 7 adult CBA/J mice undergoing unilateral cochlear implantation with serial auditory brainstem response (ABR) and distortion product otoacoustic emissions (DPOAE).

**Results:**

Implants maintained functionality for a mean of 35 days in the non-stimulated group and 19.8 days in the stimulated group. Reliable NRT and behavioral responses to electric stimulation were recorded. A robust intracochlear peri-implant tissue response with neo-ossification was seen in all cochleae. Six of seven mice maintained intact low frequency hearing up to 6 weeks following cochlear implantation.

**Conclusions:**

We demonstrate the feasibility of cochlear implantation and behaviorally significant electric stimulation in the mouse, with the potential for hearing preservation. This model may be combined with established mouse models of hearing loss and the large genetic and molecular research toolkit unique to the mouse for mechanistic and therapeutic investigations of cochlear implant biology.

## Introduction

Cochlear implants (CI) have been established as a safe and effective strategy treating severe to profound sensorineural hearing loss in both adults and children. Advances in electrode design and ‘soft’ surgical techniques have enabled combined electrical and acoustic stimulation in many patients with functional low frequency hearing, which brings improved music perception [1, 2], speech in noise understanding [3–5] and sound localization [6, 7]. The ability to implant patients with intact low frequency hearing has greatly expanded the potential CI candidate population, which by 2020 in the USA is estimated to reach 1.51 and 1.92 million people for hearing preservation (e.g. Hybrid) and conventional CIs, respectively [8].

Although advances in electrode design and speech processors have continued to improve CIs, there are still several pertinent issues that hamper their efficacy [9]. The anticipated benefits of hearing preservation over conventional CIs relies, at least in part, on preservation of functional (serviceable with hearing aid) low frequency hearing to enable combined electric and acoustic stimulation. However, a subset of patients experiences a loss of residual acoustic hearing at varying timepoints after implantation [10]. It is hypothesized that immediate post-operative hearing loss may result from insertion trauma, whereas delayed post-activation hearing loss may be secondary to multiple factors including inflammation, neurosensory cell death, strial changes or excitotoxicity associated with overstimulation [4, 11–13]. Temporal bone histopathology from a single Hybrid CI patient suggests that, in some cases, delayed residual acoustic hearing loss may be secondary to intracochlear conductive hearing losses [14] associated with the foreign body tissue response to the electrode array, consistent with previous modeling predictions [15]. Several human temporal bone histopathology series [16–21] demonstrated a comparable tissue response around the electrode array. The response consisted of inflammatory cells and areas of neo-ossification with a foreign body granulomatous reaction present in 57–96.4% of cases of conventional cochlear implantation [21]. In some [17], but not all cadaveric temporal bone series [16], the intracochlear tissue reaction, specifically the degree of neo-ossification, has been shown to negatively correlate with clinical word understanding scores. Additionally, it has been hypothesized that a peri-implant fibrotic capsule could lead to higher electrode impedances, hampering CI efficacy by lowering dynamic range and increasing power consumption [22]. Human temporal bone studies have suggested the role of a foreign body reaction in the formation of the tissue response, based upon the presence of foreign body giant cells with phagocytosed silicone or platinum implant material within the tissue sheath [19, 20]. Insertion trauma is also seen coincident with the tissue response, however it has not consistently correlated with the degree of tissue response [16, 17].

Multiple animal models of cochlear implantation have been developed to study both intracochlear and auditory pathway responses to cochlear implantation and electric stimulation. Cat [23–28], guinea pig [29–33] and rat [34, 35] CI models have facilitated study of behavioral and neural responses to acute and chronic electric stimulation in addition to providing traditional histopathological data. The guinea pig and rat models have been used by several labs for detailed examination of intracochlear changes associated with conventional and hearing preservation cochlear implantation at the gross anatomic, cellular and gene expression level [22, 36–39]. These experiments have suggested a contribution of CI insertion trauma to the intracochlear tissue response [38], described associated inflammatory gene expression changes [37, 39], established a link to electrode impedance changes [22, 28] and demonstrated the potential mitigative effects of steroid therapy [22]. Animal models have shown great utility for hypothesis generation and testing in CI biology, as well as providing a pre-clinical model for testing treatment strategies to address issues including peri-implant tissue response and loss of residual acoustic hearing.

The mouse (*Mus musculus*) has been extensively utilized for inner ear research. The ability to perform genetic manipulation and investigations in mice has yielded a variety of unique tools to aid inner ear research, including the development of transgenic reporter mice and models of genetic hearing loss [40–42]. Several etiologies of hearing loss have been modeled in the mouse, including presbycusic, noise-induced, ototoxic and genetic and their validity to the human disease state is corroborated by different objective measures including hearing tests, histologic assessment of neurosensory cell health and genetic sequencing. Despite these model species advantages, there has been a relative paucity of CI studies involving mice [43–47]. Although the overall anatomy of the human and mouse cochlea are similar, including the division of scalar partitions, the human scala tympani volume is nearly 2 orders of magnitude larger than the mouse [48]. The smaller size of the mouse cochlea makes implantation technically challenging and precludes use of human CI electrode arrays. However, the construction of a scaled mouse specific implant fabricated from materials identical to those used in human implants would allow comparable round window insertion surgical approaches and relative implant insertion depths. Implantation of a mouse specific array in validated mouse models of hearing loss could potentially be used to simulate clinically relevant CI related pathologies including intracochlear inflammation and delayed loss of residual acoustic hearing.

We have previously described an approach for passive cochlear implantation without electric stimulation [44, 46]. Inclusion of electric stimulation is relevant to any CI model, as previous data suggest that electric current flow patterns and excitotoxic overstimulation may modify intracochlear responses to cochlear implantation [11, 16]. Here, we describe a mouse model of cochlear implantation with chronic electric stimulation and the ability to use clinical CI software to obtain impedance measures, neural response telemetry (NRT) and elicit behavioral changes correlating with stimulus presentation, suggesting sensory perception of the electric stimulus. This model could be employed in future experiments utilizing the strong genetic and molecular tools unique to the mouse to investigate relevant topics of peri-implant tissue response and loss of residual hearing after CI. Additionally, in a separate set of experiments, we demonstrate feasibility of hearing preservation CI surgery in a mouse model using a comparable CI electrode array.

## Methods and materials

### Animals and study design

**Fig 1:**
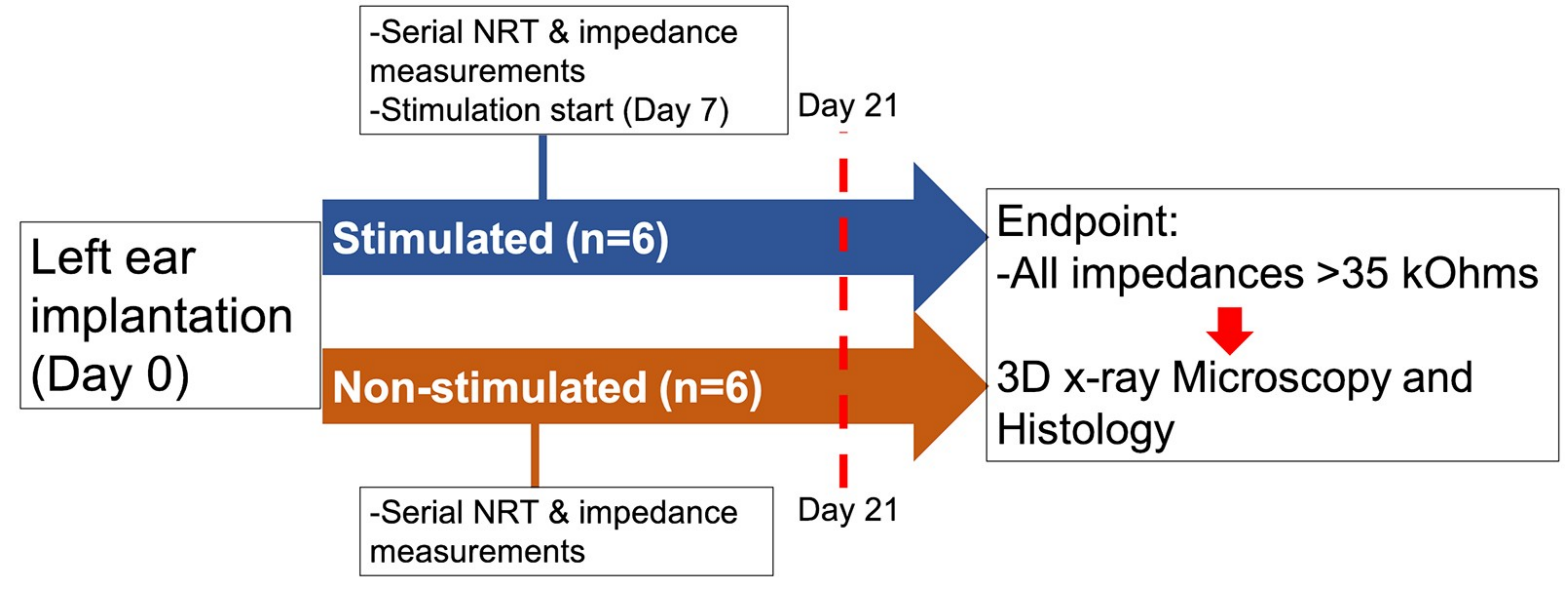
Experimental time course. Left ear cochlear implantation was performed in both the stimulated (n = 6) and non-stimulated (n = 6) groups. Serial NRT and impedance measurements began on post-operative day 7 in both groups. Electric stimulation started in the stimulated group on post-operative day 7. After post-operative day 21 and when all electrodes lost functionality (impedance > 35kOhms), cochleae were harvested for 3D x-ray microscopy and histology.

**Fig 2:**
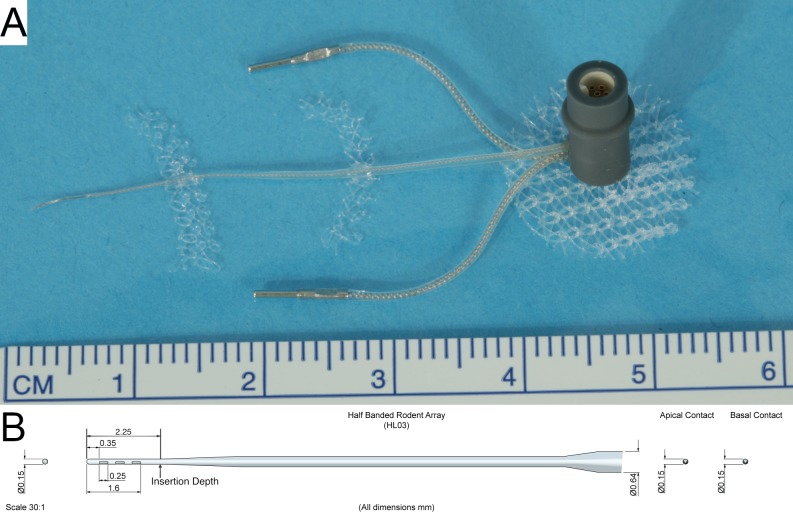
Electrode array assembly. The electrode array assembly (A) and schematic (B) are pictured. The electrode assembly consisted of a 2.25 mm long and 0.15 mm wide half-banded three contact electrode intracochlear array tapering to a wider extracochlear helixed lead wire with silicone insulation along with two extracochlear electrodes connected to a transcutaneous 6-pin connector. Modified polypropylene hernia mesh is affixed strategically to enable subcutaneous fixation and stabilization of the implant.

**Fig 3:**
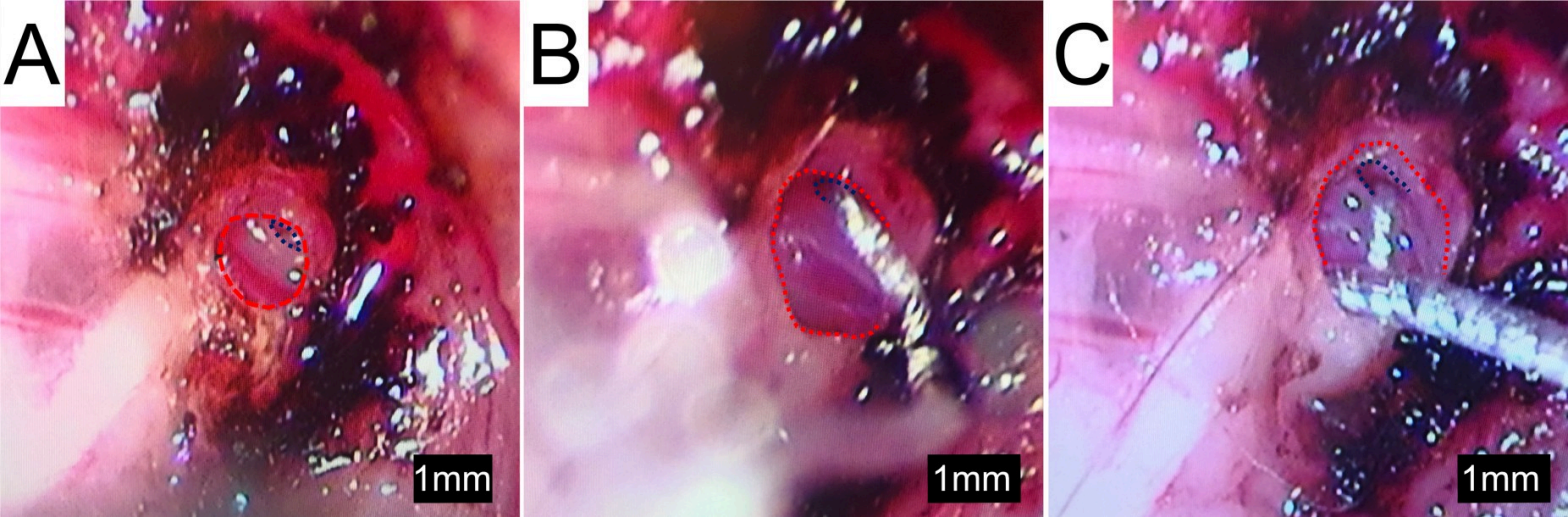
Cochlear implantation surgery. (A) Standard bullostomy (outlined in red) with exposure of the round window (outlined in blue), which is extended inferiorly in (B). After round window CI insertion, part of the implant lead wire is packed into the extended bullostomy cavity for stabilization (C).

**Fig 4:**
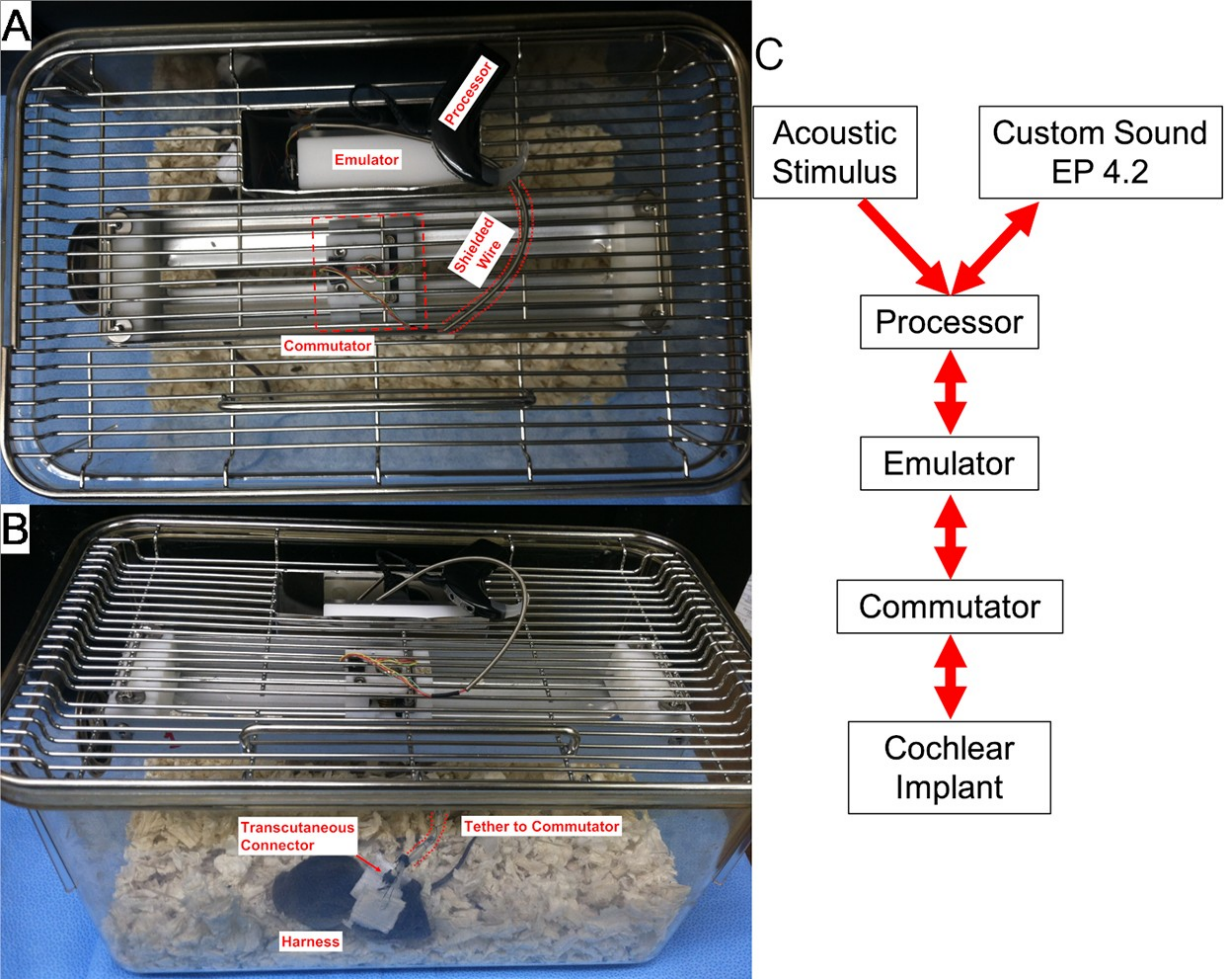
Example of the stimulation cage. (A) Top down view shows the modified cage top with the implant emulator and processor within a metal enclosure. The emulator is connected to a sliding commutator via cabling protected by a spring-shield. The commutator is connected via a short length of cable which tethers to a transcutaneous connector in the electrode array assembly, which is stabilized by a harness (B). The translating commutator allows free movement of the mouse throughout the entire caging system, while maintaining electrical connectivity. (C) Diagram of the connectivity of the system with arrows denoting the direction of stimulus or recording flow.

The study design examined the limits of a mouse model of cochlear implantation and chronic electrical stimulation in terms of animal tolerability and hardware functionality. Fig 1 shows the experimental timeline. Ten to twelve week old CBA/J mice comprised 2 groups: non-stimulated (n = 6) and stimulated (n = 6). Each group was comprised of 4 male and 2 female mice. The left ear was unilaterally implanted with a cochlear implant electrode array shown in Fig 2 (Cochlear HL03, Cochlear Limited, Australia), accurately representative of relative size and materials to clinical application. The CI electrode array was implanted via a round window insertion technique (Fig 3); the right ear served as a non-operative control. Subcutaneous Rymadyl (caprofen) (5mg/kg body weight dose; 0.3–0.4mL injection volume) and subcutaneous lidocaine (maximum dose 4mg/kg body weight; injection volume 0.04–0.05mL) were used immediately pre-operative as analgesics, with another identical dose of Rymadyl 24 hours later. Enrofloxacin (10mg/kg intraperitoneal; injection volume 0.2–0.3mL) was given for antibiotic prophylaxis. Mice were individually housed in the standard animal care facility cage, until post-operative day 7, at which point they were transferred to a custom stimulation cage (Fig 4), which integrated into the standard animal care facility housing racks and provided ad libitum access to food and water. While in the cage, a soft harness was fitted to the mouse torso to stabilize the transcutaneous connector which interfaces via a cable to a sliding commutator that connected to a CI 24RE emulator (Research device, Cochlear Limited, Australia) and sound processor (Cochlear Nucleus CP800 sound processor, Cochlear Limited, Australia); this setup allowed free movement throughout the entire cage. The total weight of the CI with transcutaneous connector (0.37g) and soft harness (1.8g) was 2.17g. Animal health including weight, grooming, wound healing and overall behavior were assessed regularly. All hardware and procedures were approved by the University of Iowa Office of Animal Resources Institutional Animal Care and Use Committee.

Starting on post-operative day 7, both groups were housed in the stimulation cage, which integrated into the standard animal care rack system, allowing ad-libitum access to water and standard food pellets placed on the cage floor. The mouse is fitted with a soft harness, which stabilizes the transcutaneous connector while it is tethered to the commutator. The commutator translates across the long-axis of the cage, providing unrestricted mouse movement within the entire cage. The commutator is connected to a CI emulator and sound processor via cabling protected by a spring-shield. The sound processor is programmed to drive the implant emulator with predefined stimulation levels and rates.

Impedances were measured daily. Neural Response Telemetry (NRT) was performed weekly. Starting 7 days post-operatively, all subjects were individually housed in custom stimulation cages. The stimulated group received electric stimulation for 5 hours, 5 days a week for 2 weeks. The non-stimulated group received no electric stimulation. We defined a functioning implant as having at least one intracochlear electrode with an impedance less than or equal to 35 kOhms, which is the upper limit permitted for stimulation programming. The primary endpoint involved two criteria. Firstly, after at least 21 post-operative days had passed and secondly, when no electrode channels (electrode) were programmable (corresponding to electrode impedances over 35 kOhms). When both criteria were met, animals were euthanized via decapitation under anesthesia with a mixture (total intraperitoneal injection volume 0.1–0.15mL) of ketamine (87.5mg/kg body weight) and xylazine (12.5mg/kg body weight) and both cochleae harvested and fixed with 4% paraformaldehyde by perfusion through the oval window, followed by overnight submersion fixation. Arrays were left in the cochlea until 3D x-ray microscopy as described below.

### Surgical technique

The surgical technique followed a modified version of the left post-auricular approach to the tympanic bulla for round window electrode array insertion described by Soken et al. [44]. After exposing the round window, an extended bullostomy (Fig 3A & 3B) was drilled to allow packing of the implant lead wire into the tympanic bulla following array placement. The round window was pierced with a 0.0025 inch sterile platinum wire followed by array insertion to a depth of 2.25 mm, such that the most proximal (basal) electrode contact was immediately out of view, past the round window and the tapered region at the distal part of the electrode rested at the round window (marked in Fig 2B). Fascia was packed around the round window to limit egress of perilymph. A short segment of the array lead was coiled in the extended bullostomy cavity and fixed with dental cement in an effort to prevent translation of any movement from the extracochlear portions of the array to the intracochlear portion (Fig 3C). A subcutaneous pocket was dissected from the post-auricular incision to the mid-thoracic spine and the transcutaneous connecter was fed through and attached via a polypropylene mesh subcutaneously with a 6–0 monocryl suture (Ethicon, USA). One extracochlear electrode was placed over each shoulder. The wound was closed with 5–0 silk suture (Ethicon, USA), which was removed 10 days post-operatively. Surgical procedures were performed under 1–3% inhalational isoflurane anesthesia.

### Cochlear implant electrode array assembly

The CI electrode array consisted of a silicone carrier with 3 half-banded platinum electrode contacts. The intracochlear portion measures 2.25mm in length and 0.15mm in diameter, tapering to 0.3mm in the extracochlear portion (Fig 2). The lead wires are helixed to provide strain relief. The array lead and 2 extracochlear electrodes are attached to a 6-pin connector (Nanocircular, Omnetics, USA) insulated by a polypropylene housing. The electrode assembly can be connected to an external stimulation source (Fig 4C).

### NRT and impedance measures

NRT and impedance measurements were made using clinical programming software (Custom Sound EP, Cochlear Custom Sound Suite 4.2 Programming software, Cochlear Limited, Australia). The electrodes were not preconditioned prior to measurement to accurately reflect the procedures used clinically with hearing preservation cases; impedance measurements were conducted in monopolar (MP) 1 + 2 mode, which measures the electrical impedance between one intracochlear electrode (active terminal) and both monopolar extracochlear electrodes shorted together (counter terminal). NRT was measured using the Auto-NRT detect feature of the programming software using the Probe-Masker paradigm. The intracochlear electrode with the lowest impedance at the time was chosen as the active probe (stimulating) electrode and the intracochlear electrode with the next lowest impedance served as the active recording electrode; MP 1 and 2 served as the indifferent probe and recording electrodes, respectively. A charge balanced, biphasic square stimulus level series starting at an amplitude of 89 μA (90 Current Levels (CL); 2.22 nC/phase) and increasing to an amplitude of 377 μA (170 CL; 9.43 nC/phase) or until compliance limits are reached, was presented to determine an NRT threshold. The stimulus probe was presented with 25 μs pulse width and 7 μs interphase gap, at a probe rate of 80 Hz, with 50 sweeps. Recording was performed with a 98 μs recording delay, at a sampling rate of 20492 samples per second with 32 samples per stimulus sweep. The masking procedure was performed with the same probe electrode configuration as the active probes, at a masking current level 10 CL above the active current level with a 25 μs pulse width, 7 μs interphase gap, at a rate of 100 Hz with a 400 μs masker interval. The current level (CL) unit represents the amplitude of the current pulse on a log scale. Within the parameters of the 24RE CI emulator used for this study, 0 CL corresponds to 17.5 μA and 0.44 nC/phase and 255 CL to 1750 μA and 43.75 nC/phase.

### Chronic stimulation

The CI processor was programmed with Custom Sound EP (Cochlear, Australia). All functioning intracochlear electrodes were shorted together via a software patch to effectively reduce impedance across the system and allow uniform stimulation amongst subjects. Volume sensitivity was set to maximum. The threshold and comfort (T&C) levels were matched at 30 CL below NRT threshold, ensuring a constant level of stimulation without dynamic range or amplitude modulation. This stimulus level was chosen to avoid any stimulation that may cause discomfort and adverse behavioral changes. The maximum sensitivity and maximum volume settings were selected within Custom Sound Programming Suite 4.2 (Cochlear, Australia), which sets the acoustic noise threshold to evoke a pulse train as 10 dB SPL. The programmed processors were connected to the implant emulator and connected to the electrode array assembly via the stimulation cage for 5 hours, 5 days a week. The stimulation CL was adjusted according to shifting NRT threshold. Stimulation history logs were checked to ensure the ambient animal housing noise levels drove the processors to deliver constant stimulation during the experimental hours.

### In-vivo implant imaging

In-vivo intracochlear positioning of the electrode array was confirmed on post-operative day 7 via x-ray. X-ray was also used to investigate device integrity and positioning following sudden changes in electrode impedance or NRT threshold. Images were obtained on a Carestream MS FX Pro (Bruker, USA) with magnification stage at 35 KVP and 149 μA with 0.4 mm filtration and an exposure time of 40 seconds, producing pixel sizes of 42.6 microns. Animals were sedated with an anesthetic mixture (total intraperitoneal injection volume 0.1–0.15mL) of ketamine (87.5mg/kg body weight) and xylazine (12.5mg/kg body weight) during in-vivo imaging.

### 3D x-ray microscopy

Fixed cochleae were prepared for non-destructive 3D x-ray microscopy with array in-situ. Images were obtained using a Zeiss Xradia Versa 3D x-ray Microscope (Zeiss, USA) with a voxel size ranging 0.65–3μm. Three dimensional reconstructions and artifact rejection were implemented within Scout and Scan Control System and XM Reconstructor–Cone Beam 10 software (Zeiss, USA). Image viewing and 3D reconstruction were conducted within Visual SI (ORS, Canada). An initial image was obtained with the array in-situ. The array was subsequently removed in a slow, controlled manner. Tissue was osmicated to enhance soft-tissue contrast. The cochlea was rinsed with 0.1M phosphate buffer. 1% osmium tetroxide with 1.5% potassium ferricyanide was gently perfused through the scalae and then immediately immersed in a 1% osmium tetroxide with 1.5% potassium ferricyanide solution and placed on rotator for 2 hours. Cochleae were then rinsed with 0.1M phosphate buffer solution and returned for imaging. The cochleae underwent repeat 3D x-ray microscopy and the resulting image series was merged with the previous array in-situ image series to obtain projections of the array within the cochlea.

3D volume segmentation of the scala tympani was performed on 3D x-ray microscopy cochlea image stacks obtained after implant removal and specimen osmification. A gaussian filter was applied to image stacks prior to manual volume segmentation in Dragonfly software (ORS, Canada). This procedure was performed by a single laboratory staff blinded to group. Raw volumetric data for the scala tympani, implant tract, neo-ossification and soft tissue response was recorded. Fractional volumes of the neo-ossification and soft tissue response were calculated by dividing the volume of the respective region of interest by the total volume of the scala tympani.

### Histology

Following 3D x-ray Microscopy, osmicated cochleae are decalcified in 0.1M EDTA (pH 7.5). Cochleae are then dehydrated with graded alcohols. Plastic infiltration of cochlea is accomplished using a low viscosity embedding media Spurr’s kit (cat. 14300 Electron Microscopy Sciences). Before embedding in the epoxy resin, the cochlea is divided in the mid-modiolar plane by making a perpendicular cut through the round window and the apex. The halves are then placed in a block mold filled with Spurr’s resin and polymerization is achieved in an oven set at 75 degrees Celsius.

1 μm thick sections are cut using a Reichert Ultramicrotome OmU3 (Reichert, Austria) and mounted on slides. Sections are then stained by using a Richardson’s staining method, covered slipped, examined and photographed with the 4X objective using a Nikon microscope with a Coolpix S10 digital camera (Nikon, Japan) attached with a MM99 Martin Microscope Company adapter (Martin Microscope Company, USA).

### Hearing preservation surgery

A separate study was performed to determine the rate of hearing preservation in mouse cochlear implantation and observe for delayed hearing changes over a 6-week period in those with intact hearing following surgery. Successful hearing preservation was defined as less than or equal to 15 dB auditory brainstem response (ABR) threshold shift at 8 and 16 kHz. Seven (n = 7) 10–12 week old adult CBA/J mice underwent unilateral left cochlear implantation, 5 males and 2 females. The implanted array was identical to that described above except for the absence of extracochlear return electrodes and an alternative lead wire design. The surgical technique was similar to that described above with a 2.25mm depth of insertion, except with the additional use of cyanoacrylate glue and 9–0 nylon suture (Covidien, USA) to stabilize the implant near the bulla instead of dental cement. No electric stimulation was performed.

Hearing was assessed by ABR and distortion product otoacoustic emissions (DPOAE) at pre-operative baseline and at 2, 4 and 6 weeks post-operatively in the left (experimental) ear. Right ear ABR and DPOAE was only performed at baseline and 6 weeks post-operatively to reduce total anesthetic burden. ABR and DPOAE was performed as previously described [46]. Briefly, ABR was recorded in response to 8, 16 and 32 kHz tone-bursts. ABR threshold shift was calculated from the difference between the respective post-operative day and baseline values. DPOAE was measured in response to f2 stimuli of 4000, 5657, 8000, 11314, 16000, 22627 and 32000 Hz, maintaining a frequency ratio of f2/f1 = 1.22. f1 and f2 stimulus amplitudes were fixed at 65 and 55 dB SPL. Animals were anesthetized with an anesthetic a mixture (total intraperitoneal injection volume 0.1–0.15mL) of ketamine (87.5mg/kg body weight) and xylazine (12.5mg/kg body weight) during ABR and DPOAE procedures.

### Analysis

All statistical analysis, where mentioned, was performed with GraphPad Prism software (GraphPad Software, USA). Impedance data were analyzed via two-way ANOVA with repeated measures. Two-tailed t-test was used to analyze NRT and 3D x-ray volume segmentation data. ABR and DPOAE data were analyzed via one-way ANOVA with repeated measures with follow-up of significant effects with pairwise multiple comparisons via Holm-Sidak test. Statistical significance was set at an alpha level of 0.05 (p<0.05).

## Results

### Animal tolerability

Animals tolerated surgery well, with the exception of 2 cases of intra-operative mortality related to stapedial artery injury and 4 cases of early intra-operative complications related to anesthesia. Additional animals were used to reach study subject number goals. We found that low dose 1–3% isoflurane inhalational anesthesia reduced peri-operative mortality compared to injectable ketamine and xylazine. Animal feeding and grooming habits were not inhibited by the surgery and all animals maintained at least 90% of their pre-operative body weight; the animals did not show any alteration in their normal habits or ability to move within the cage. No signs of vestibular dysfunction, including abnormal gait or circling behavior were observed. Electric stimulation did not alter any of the aforementioned observations.

### Impedance measures and implant functionality

**Fig 5:**
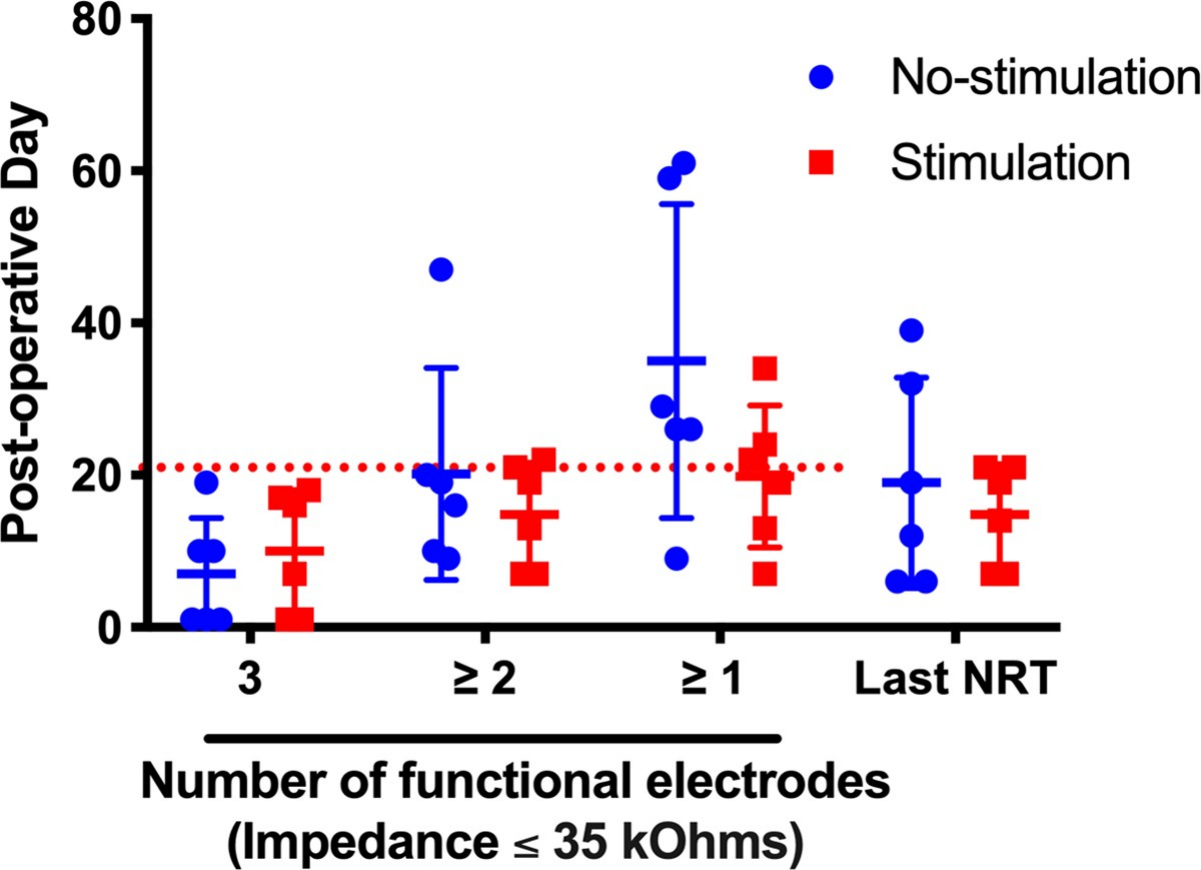
Impedance and NRT over time. Mean and individual duration of electrode functionality (impedance ≤ 35 kOhms) and time of last obtainable NRT recording. The left side of the figure portrays the duration that each implant maintained the respective number of functioning electrodes. The right side of the figure portrays the last timepoint at which an NRT response could be obtained for each implant. Non-stimulated subjects are represented by blue circles and stimulated subjects by red squares. Horizontal bars represent mean group values. The red, dotted line marks the 21 day experimental endpoint threshold. 5/6 and 3/6 subjects in the no-stimulation and stimulation groups met or surpassed the 21 day threshold of maintaining at least one functional electrode, respectively. NRT responses were generally obtainable while at least 2 electrodes maintained impedance levels ≤ 35 kOhms. Error bars represent Standard Deviation.

**Fig 6:**
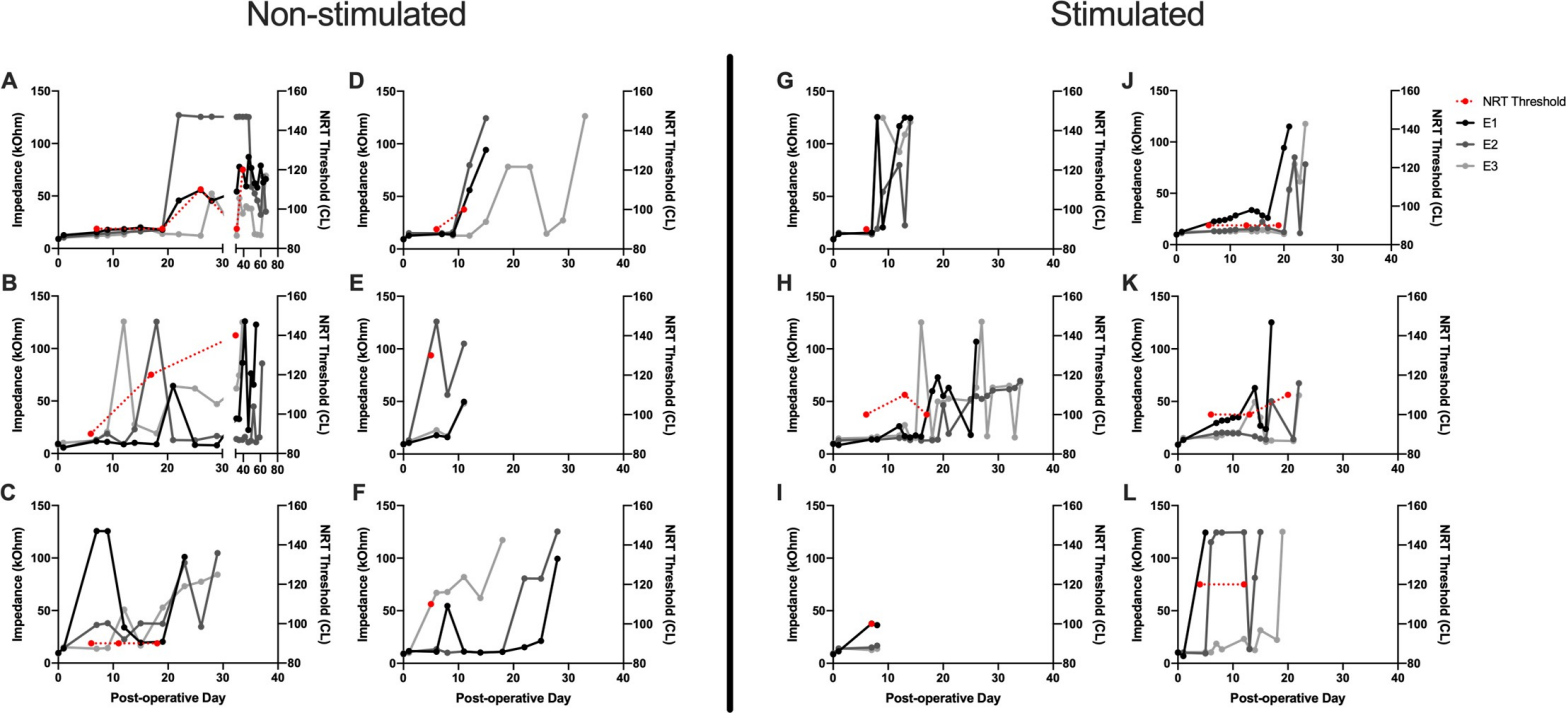
Serial impedance and NRT threshold values over time. Individual subject impedance values per electrode and NRT threshold are plotted over time. A-F represent individual data for the non-stimulated subjects and G-L for the stimulated subjects. Day 0 represents impedance values obtained immediately prior to implantation. NRT threshold values (red) are scaled to the right sided y-axis. Impedance values are in grayscale and scaled to the left sided y-axis. The most basal electrode (E1) is black, middle electrode (E2) dark gray and most apical electrode (E3) light gray. Impedance levels of 125 kOhms represent the measurement limit of the system.

At the onset of the study, a functioning electrode was defined as having an impedance less than or equal to 35kOhms based upon the parameters of Clinical CI programming software (Custom Sound EP, Custom Sound Programming Suite 4.2, Cochlear, Australia), which defines this as the impedance threshold below which an electrode can be programmed. Fig 5 shows the duration that implants maintained 1, 2 or 3 functioning electrodes. Mean duration until loss of one or two electrodes was 7 and 20.2 days for the non-stimulated group and 10 and 14.8 days for the stimulated group. The non-stimulated groups maintained at least one functioning electrode for a mean of 35 days versus 19.8 days in the stimulated group. Analysis did not reveal any significant difference between groups for the duration that they maintained 1, 2 or 3 functional electrodes. There was considerable variability in this outcome as seen in the individual impedance plots, with 5/6 of the non-stimulated and 3/6 of the stimulated subjects maintaining at least one functional electrode at post-operative day 21 (Fig 6). Although most electrodes show a trend toward gradual impedance increase, some electrodes show sharp increases to 125 kOhms, the measurement limit of the system and likely corresponds to an open circuit. We hypothesize the event of sharp impedance rise is attributable to an open circuit due to fatigue failure of the lead wires within the CI electrode array, as was seen during x-ray imaging.

### Neural response telemetry and behavioral responses

**Fig 7:**
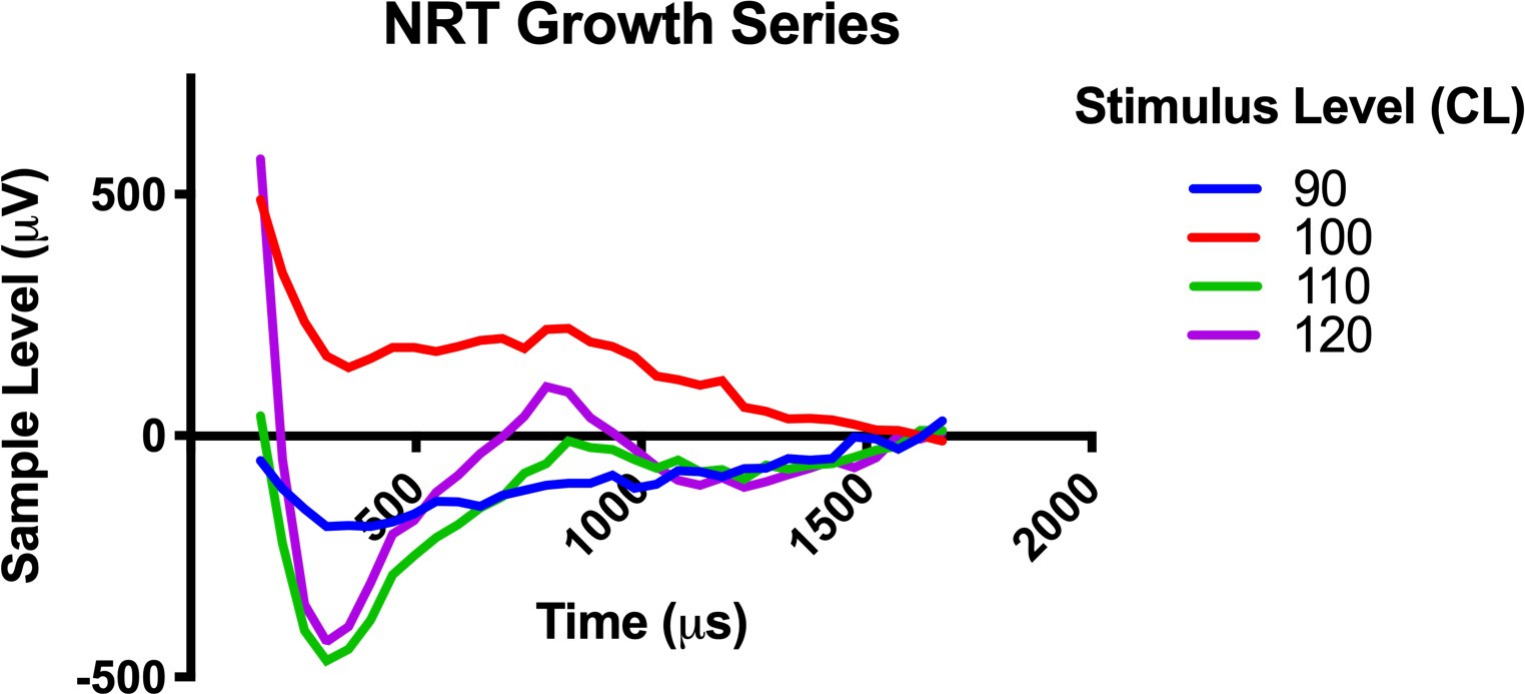
Neural response telemetry (NRT) growth series. Individual NRT responses in response to an escalating stimulus level between 90 and 120 CL is presented as an example of a typical NRT growth series.

NRT threshold was determined by visual confirmation of the lowest stimulus level at which a N1 and P2 peak response could be seen, which generally correlated with the threshold obtained from the software threshold auto-detect feature. Fig 7 shows a typical NRT growth series with a threshold of 100 CL. The N1 nadir is seen near 300μs with the P2 peak near 800μs. Fig 6 shows the serial NRT threshold values for the lowest recorded threshold value on the respective day. NRT threshold values ranged 90-140CL. NRT responses were detectable up to a mean of 19 days in the non-stimulated group and 14.8 days in the stimulated group; this difference was not significant (Fig 5). The duration that implants maintained a detectable NRT response matched the duration that at least 2 electrodes were functional, since NRT requires at least 2 functioning electrodes for stimulus presentation and recording.

Behavioral responses to electric stimulation at NRT threshold were consistently seen in non-sedated subjects. In response to a 30 second stimulus train at NRT threshold, animals were typically observed to have distinct changes in grooming habit corresponding with the offset / onset timing of the stimulus. The most common response was a temporary pause in grooming or feeding during the duration of the stimulus. No adverse signs of pain or discomfort were seen during these tests, including erratic behavior or circling. Chronic stimulation at 30 CL below NRT threshold did not produce any durable changes in normal behavior during the 5 hour stimulation blocks. Stimulation usage logs from the CI processor confirmed that the implant was stimulated for the entire 5 hour stimulation block.

### In-vivo x-ray imaging

**Fig 8:**
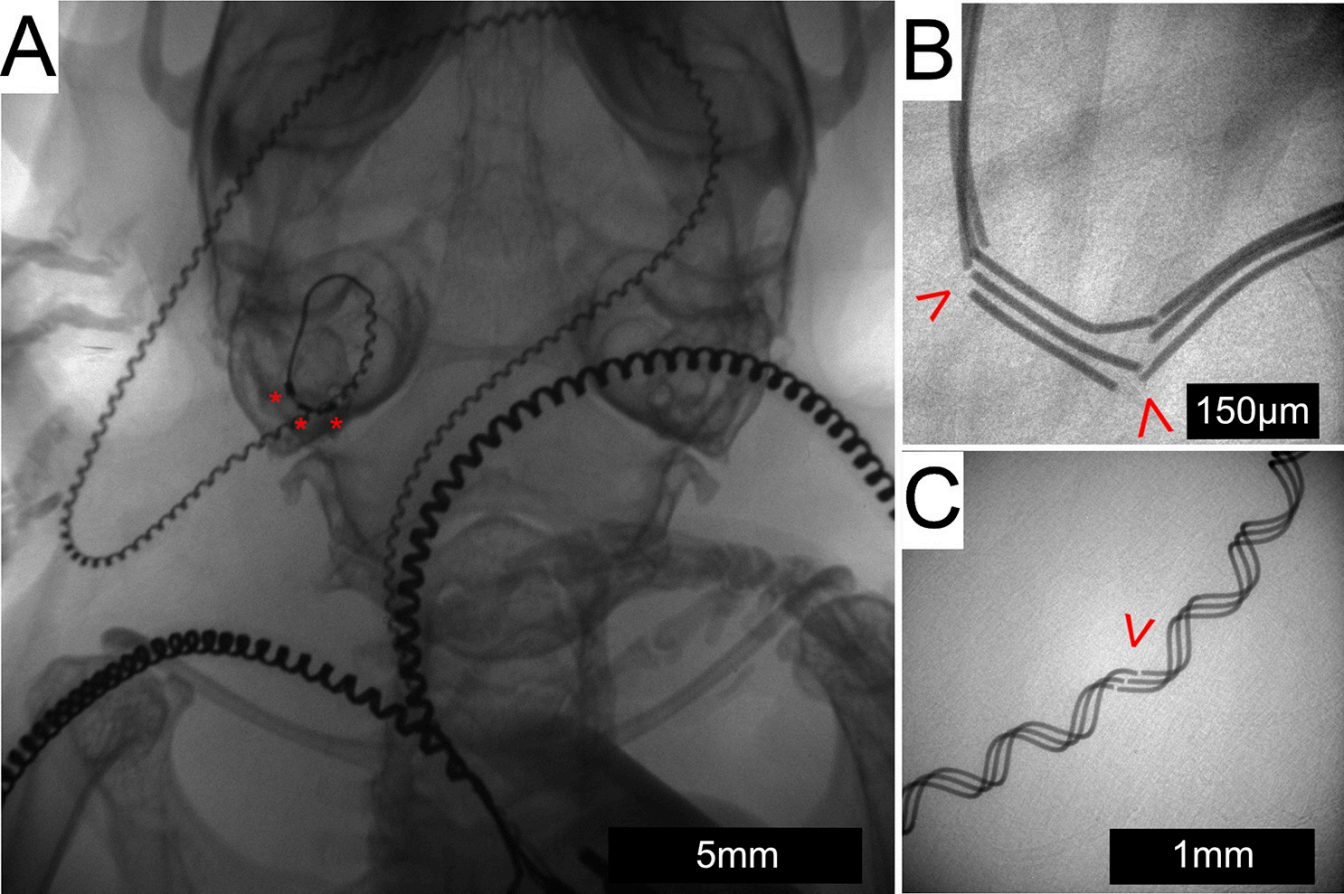
In-vivo x-ray imaging. X-ray imaging shows an intracochlear position of the array in the left cochlea of a live subject with the electrode contacts labeled by a red “*” (A). The lead wire can be seen traveling across the posterior base of the skull to meet the 2 extracochlear electrode leads as they head toward the connector located on the back (not seen in this image). Fractures (red arrowhead) occurred in both the straight (B) and helixed portions (C) of the array lead wire.

X-ray was used to assess intracochlear array position and to investigate the integrity of the electrode array lead or malposition following abrupt changes in electrode impedance. Fig 8A shows typical intracochlear positioning of all 3 electrode contacts, which was present for all subjects on post-operative day 7. All subjects maintained stable intracochlear positioning of all 3 electrode contacts until the study endpoint except for one stimulated subject, which showed extrusion of the entire implant from the cochlea on post-operative day 27.

Fracture of the array lead wires were found in conjunction with abrupt impedance changes. Fractures were observed within the straight portion of the lead wire (Fig 8B), adjacent to the intracochlear electrodes and in the more proximal helixed portion (Fig 8C). Fracture typically occurred between the outside of the tympanic bulla and mouse neck, which represents a region of high cyclic strain secondary to mouse head movements. Fractures were observed in 4 subjects per group, summarized in Table 1. No fractures were observed in the wires of the extracochlear electrode leads.

**Table 1:** In-vivo x-ray implant outcomes.

	Wire fracture (straight)	Wire fracture (helixed)	Intact	Extruded
Non-stimulated	2	2	2	
Stimulated		4	1	1

### 3D x-ray microscopy and histology

**Fig 9:**
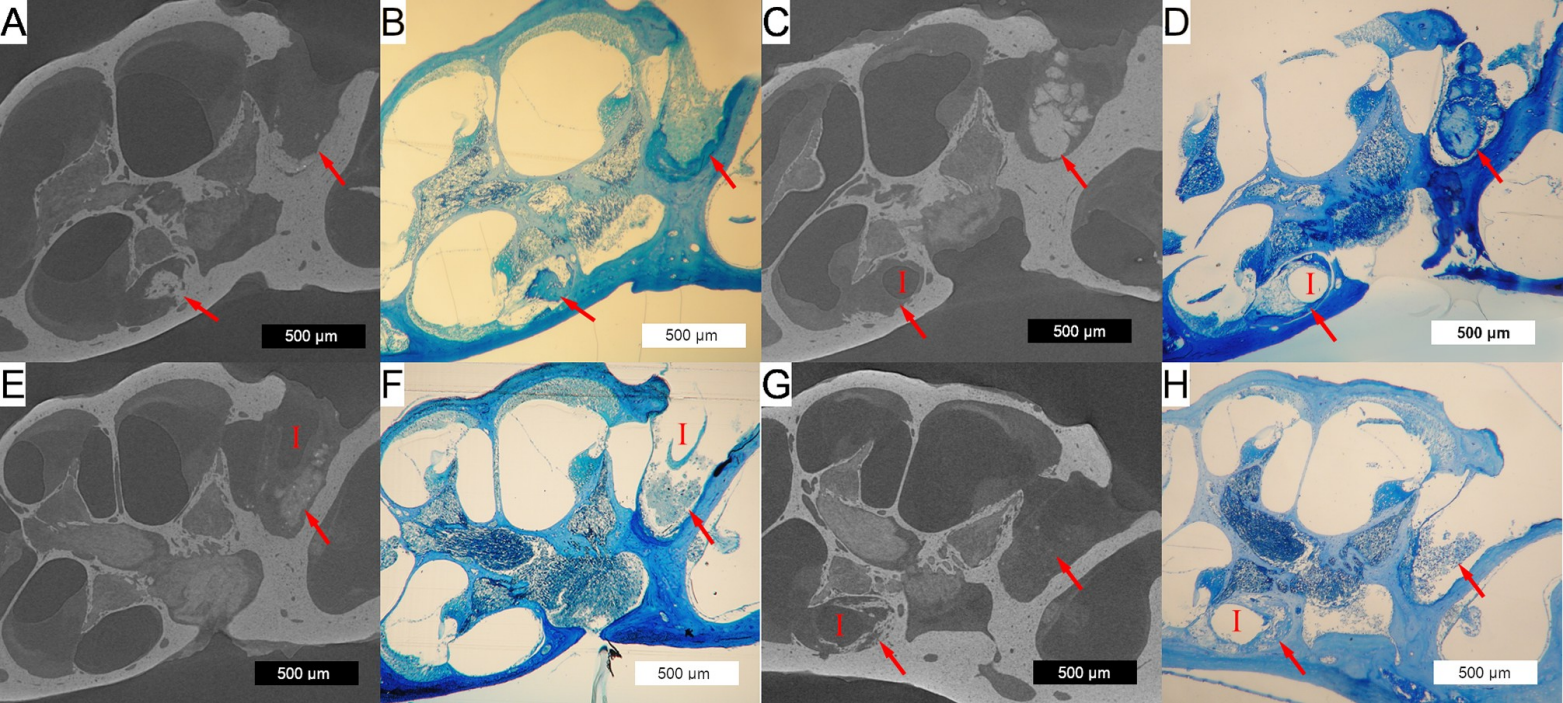
3D x-ray microscopy and histologic co-localization. 3D x-ray microscopic images (A,C,E&G) are co-localized next to corresponding histologic sections (B,D,F and H) for non-stimulated (A-D) and stimulated (E-H) subjects. The implant tract (marked by a red “I”, where visible) with adjacent soft tissue and neo-osteogenesis is seen in the round window and basal turn of the scala tympani. Representative 500μm scale bars are provided for each image. Red arrows mark the tissue response within the scala tympani and round window area.

Fig 9 shows typical histologic and 3D x-ray microscopy findings in both non-stimulated (A-D) and stimulated (E-H) subjects. 3D x-ray microscopy images are oriented to corresponding mid-modiolar histologic sections from the same subjects. The soft tissue detail of the histologic sections is complemented by the bony detail and non-destructive process of 3D x-ray microscopy. The non-destructive nature of 3D x-ray microscopy is highlighted in Fig 9C & 9D, showing partial fracture of the otic capsule as an artifact of histologic sectioning (D), which is intact in the 3D x-ray microscopy image (C).

A robust tissue response, including areas of neo-ossification was seen throughout all implanted cochleae in both groups, extending from the round window to an area immediately distal to the tip of the electrode array. This finding was confined to the scala tympani, with no tissue response seen within the scala media or scala vestibuli. An obvious implant tract could be seen in all subjects, with variable positioning of the electrode along the lateral wall to modiolus axis. No obvious signs of basilar membrane penetration or fracture of the osseous spiral lamina or modiolus were present.

**Fig 10:**
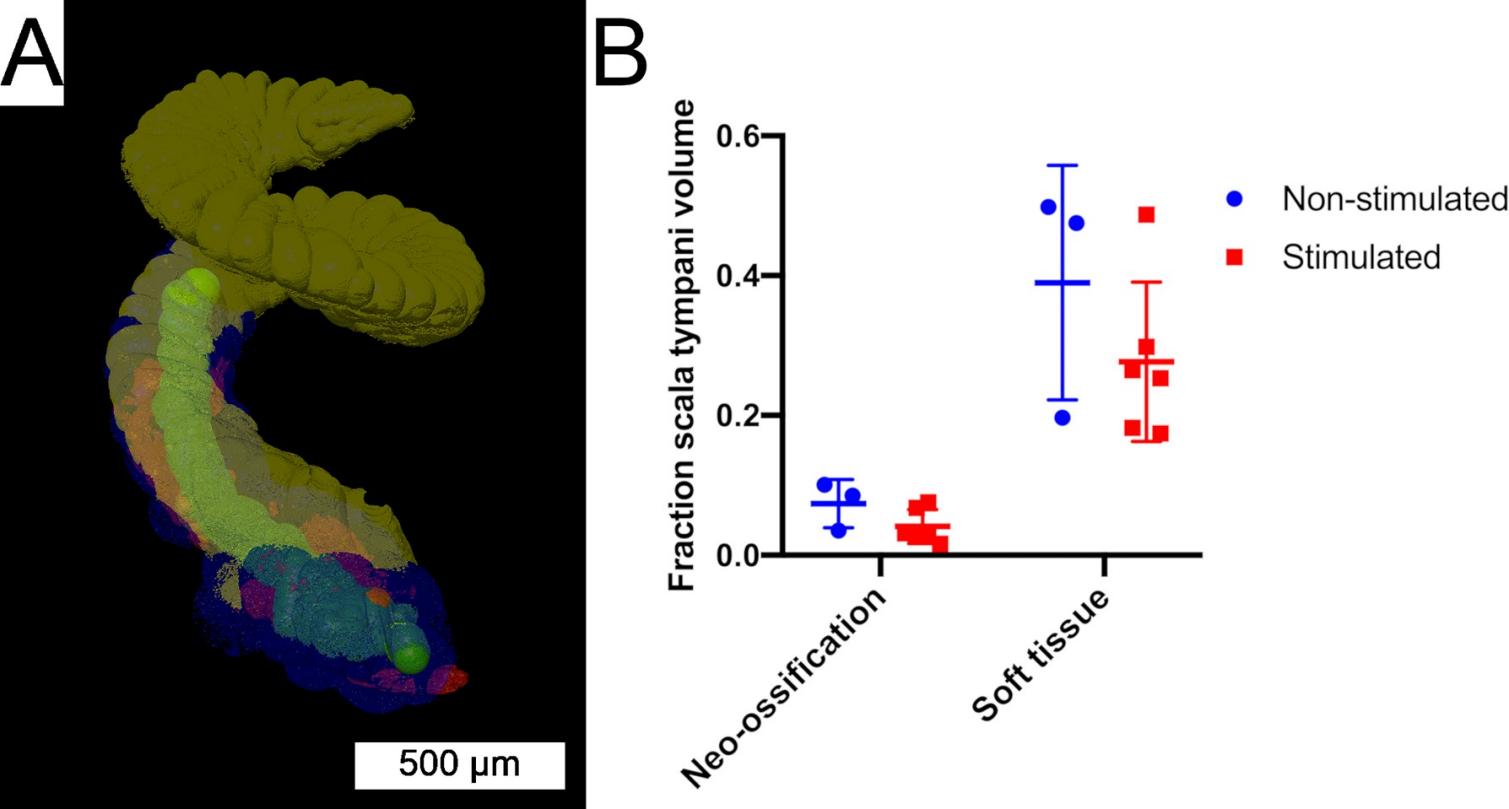
Volumetric cochlea segmentation. (A) 3D rendering of a volume segmented 3D x-ray microscopy cochlea image stack. Regions of interest include the implant tract (green), soft tissue response (blue), neo-ossification (red) and unoccupied scala tympani (yellow). (B) The fractional volume of the scala tympani occupied by neo-ossification or soft tissue response was quantified. Individual data for non-stimulated (blue dots) and stimulated (red squares) subjects are include with horizontal bars showing group mean values. There were no significant differences between groups (p>0.5). Error bars provide SD.

Segmentation of 3D x-ray microscopy image stacks allowed volumetric quantification of the post-implantation inflammatory response within the scala tympani (Fig 10). This analysis was restricted to image series obtained from cochleae without the implant left in-situ, which included n = 3 non-stimulated and n = 6 stimulated specimens. Mean volume of the scala tympani across all specimens was 0.449 μL. Mean fractional volume of the scala tympani occupied by neo-ossification was 0.0737 (7.37%) and 0.0411 (4.11%) for the non-stimulated and stimulated groups, respectively. Mean fractional volume of the scala tympani occupied by the soft tissue response was 0.389 (38.9%) and 0.276 (27.6%) for the non-stimulated and stimulated groups, respectively. There were no significant differences between groups for the fractional volume of neo-ossification (p = 0.139) or soft tissue response (p = 0.277).

**Fig 11:**
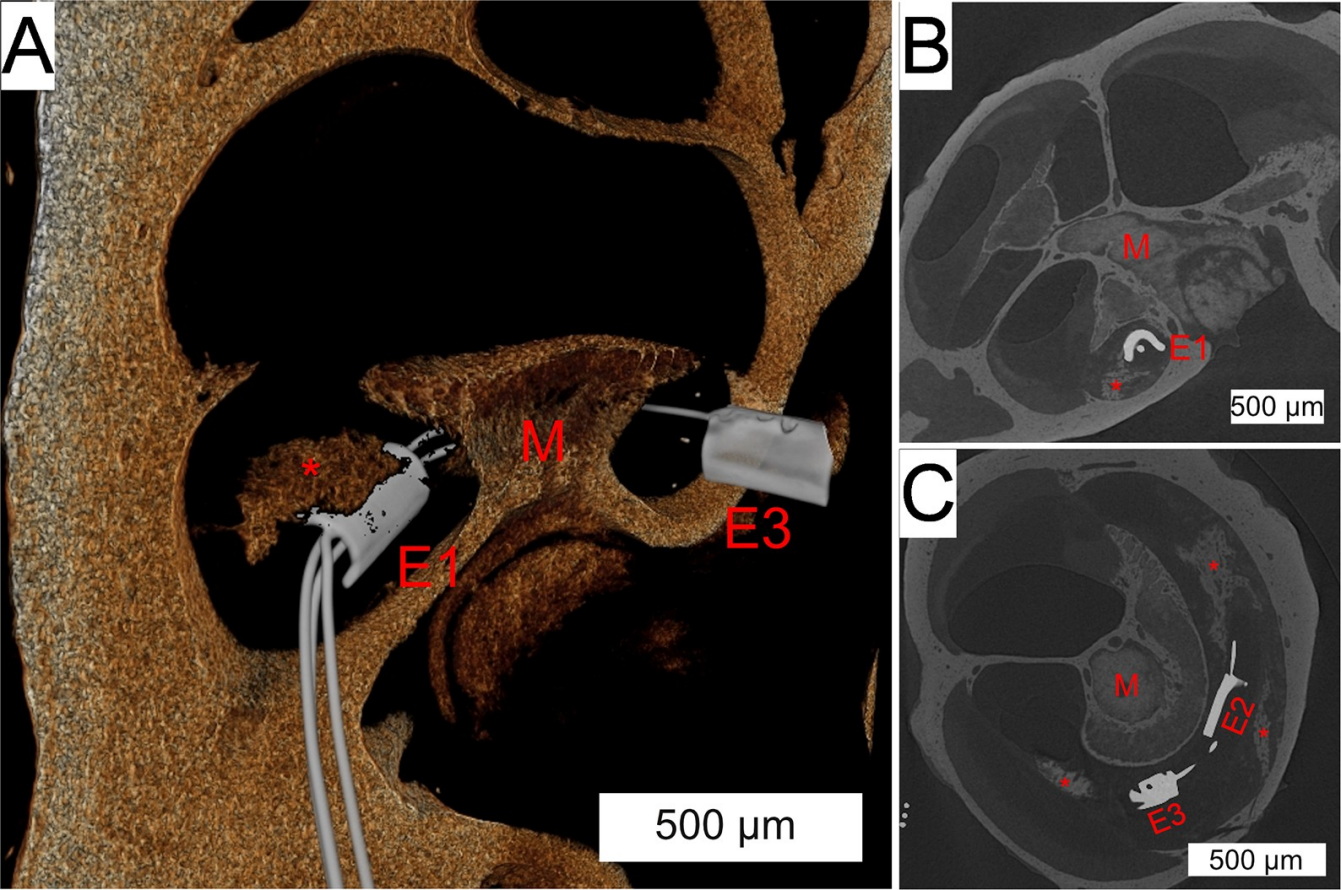
Co-registration of electrode array within 3D x-ray microscopic images. (A) 3D reconstruction of a 3D x-ray microscopic image shows the array trajectory through the scala tympani, around the modiolus (red “M”). The most apical electrode (red “E3”) and most basal electrode (red “E1”) are seen with a peri-modiolar orientation, with the middle electrode obscured by the modiolus. The red “*” denotes a foci of peri-implant neo-osteogenesis. Coronal (B) and axial (C) sections show the intrascalar orientation and trajectory of the implant with multiple areas of neo-osteogenesis (red “*”). The most basal (red “E1”), middle (red “E2”) and apical (red “E3”) are seen.

Merged images of 3D x-ray microscopy series were obtained with and without the array in-situ (Fig 11). Consistent bony landmarks visible in both image series allowed co-registration of the images, allowing reduction of implant associated artifact obscuring image detail in the immediate vicinity of the electrodes. This technique allows confirmation of modiolar oriented electrode positioning, as well the orientation of the local neo-ossification relative to the position of the electrodes.

### Hearing preservation

**Fig 12:**
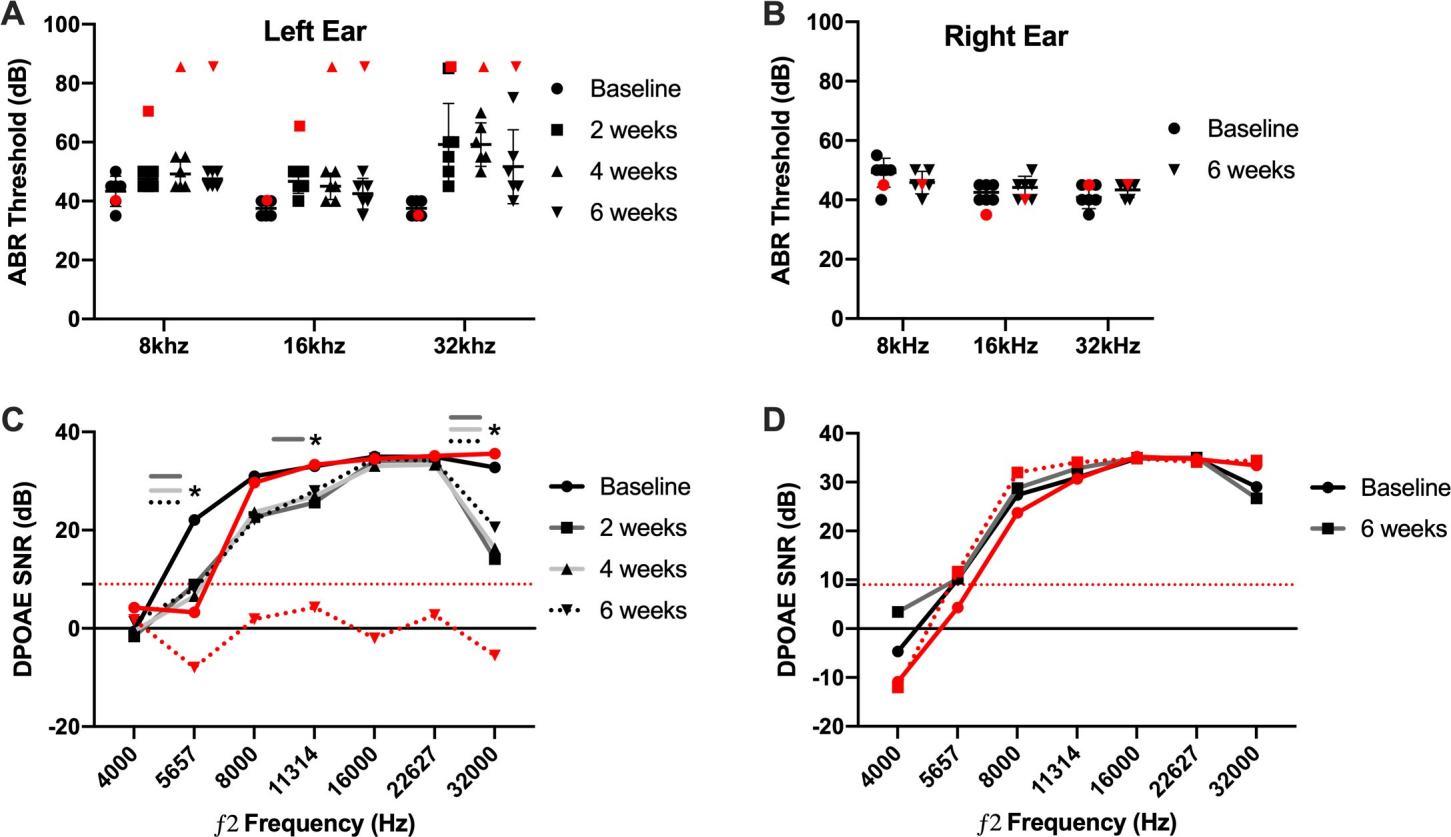
ABR and DPOAE measures of hearing preservation. Left, implanted (A) and right, non-implanted (B) ear ABR thresholds are plotted by individual subject with representative black symbols according to testing day. The black horizontal bars represent mean values. Red symbols indicate the 1 subject excluded secondary to significant hearing loss post-implantation. ABR thresholds maintained within 15 dB SPL of baseline values for all experimental subjects (black symbols) at the 8 and 16 kHz frequencies. There were no significant differences between timepoints at any frequency (p>0.05). Error bars represent SD for the 6 subjects meeting hearing preservation criteria. (C) and (D) show mean DPOAE signal to noise ratio (SNR) for the left (implanted) and right (non-implanted) ears, respectively. ‘*’ denotes statistically significant (p>0.05) difference from baseline SNR values at respective timepoints. Within our system, a SNR ≥ 9 is considered an intact DPOAE; this threshold is denoted by the red, dotted horizontal line. The solid red line represents baseline values for the 1 excluded subject and the dotted red line represents final 6-week DPOAE SNR values.

In a separate study examining hearing preservation in mouse cochlear implantation we applied inclusion criteria of successful hearing preservation as ≤ 15 dB ABR threshold shift at the 8 and 16 kHz frequencies at 2 weeks post-operatively. Within these criteria, 6/7 or 85.7% of subjects had successful hearing preservation after cochlear implantation. The one subject not meeting the hearing preservation criteria (red data points in Fig 12) experienced ABR threshold shifts of 30, 25 and 50 dB at 8, 16 and 32 kHz by post-operative week 2 as well as a concurrent loss of DPOAE response. Additionally, further hearing loss was noted at 4 and 6 weeks post-operatively by ABR measurements. This subject was excluded from the additional analysis as the intent was to study hearing stability over time with successful initial hearing preservation cochlear implantation.

All subjects experienced ≤ 10 dB ABR threshold shifts at 8 and 16 kHz at all timepoints in the implanted ear (Fig 12A). Mean ABR threshold shift at 32 kHz was 21.67 dB at post-operative weeks 2 and 4, decreasing to 14.17 dB by post-operative week 6. There was no significant effect of testing day at any frequency. Right ear controls experienced no significant change in ABR threshold over the course of the study (Fig 12B).

DPOAE SNR > 9 dB was considered a detectable response and is noted by a horizontal red dotted line in Fig 12C & 12D. In the implanted ears, the mean SNR was > 9 dB in all instances except for the f2 frequencies of 4000 at all timepoints and 5657 Hz on all post-operative timepoints. An effect of testing day was seen in the 5657 (p = 0.001), 8000 (p = 0.037), 11314 (p = 0.022) and 32000 Hz (p = 0.004) ƒ_2_ frequencies. Post-hoc pairwise comparisons at these 3 frequencies demonstrated significant differences between the DPOAE SNR at baseline compared with post-op weeks 2, 4 and 6 at 5657 Hz (p<0.01), post-op weeks 2 at 11314 Hz (p<0.05) and post-op weeks 2 and 4 at 32000 Hz (p<0.05); other pair-wise comparisons were non-significant (p>0.05).

## Discussion

This study demonstrates the technical feasibility of mouse cochlear implantation with chronic electric stimulation, and the ability of hearing preservation surgery. Overall, 8/12 subjects maintained one or more functioning electrodes for at least 3 weeks, enabling electric stimulation over the same time period. Behavioral responses to electric stimulation and objective measures (NRT) evidenced successful stimulation of the mouse auditory system. Further, the surgery and experimental procedures were well tolerated by mice without any signs of vestibulopathy or implant related complications.

An individual electrode impedance ≤ 35 kOhms is required for programming of electric stimulation within the Clinical CI programming software (Custom Sound EP, Custom Sound Programming Suite 4.2, Cochlear, Australia). This level represents the threshold for which electric stimulation can safely be administered without theoretical risk of tissue damage based on the Shannon equation [49]. Impedance measurements were followed on all electrodes until reaching this failure threshold. Different patterns of impedance change occurred among subjects and between electrodes on the same implant. Several electrodes showed a sharp impedance rise from ≤ 35kOhms to 125kOhms, which represents the measurement limit of the system and corresponds to an open circuit value. This pattern could be attributed to the lead wire fractures seen co-occurring temporally on in-vivo x-ray in specific cases, which would create the open circuit. The presence of fractures may also explain the fluctuating pattern of impedance in some electrodes, wherein the fractured ends of the lead wire may temporarily re-approximate with mouse repositioning, bridging the previously open circuit. It is possible other unidentified hardware malfunctions could also have contributed to impedance changes in some cases. Other electrode impedance profiles showed a slower, gradual increase in impedance overtime until the failure threshold was reached. It is possible that this pattern could represent accumulation of new soft tissue and neo-ossification around the implant as part of an inflammatory response, similar to that seen in other model systems [22, 28]. The non-stimulated group showed a trend of longer lasting electrode functionality compared to the stimulated group, although this was not statistically significant. Although this study was not designed to determine such a cause, we hypothesize this finding could be secondary to sub-clinical changes in animal behavior with stimulation that introduced further physical strain on the implant or alteration of the intracochlear tissue response from stimulation, which modified electrode impedance values. Future studies will be needed to investigate these hypotheses.

The NRT procedure measures the electrically evoked compound action potential (eCAP) response of the 8^th^ cranial nerve [50] and was used to confirm stimulation of the auditory system and guide electrode mapping. The procedure requires at least 2 functioning intracochlear electrodes for stimulation and recording. Following this principle, NRT responses were detectable if there were at least 2 functioning electrodes. NRT response thresholds detected via the AutoNRT feature [51] in Clinical CI programming software (Custom Sound EP, Custom Sound Programming Suite 4.2, Cochlear, Australia) correlated well with those visually confirmed by the experimenter in the presence of an N1 and P2 peak morphology. To our knowledge, these data represent the first intracochlear eCAP recordings from a cochlear implant in the mouse. The mouse NRT threshold range of 90–140 CL (89–219 μA) found in this study corresponds with a similar range found in a rat CI model [35]. Interestingly the N1 and P2 latencies and overall waveform morphology were similar to those seen in human subjects. Behavioral responses, including abrupt changes in grooming or feeding habits corresponding to the onset and offset of the stimulus, were typically seen at NRT threshold level. Based on prior human studies [52, 53], electrodes were mapped with both T&C levels at 30 CL below NRT threshold, to ensure the stimulus was below the true C level. No dynamic range (T equals C level) was programmed to normalize the intensity of electric stimulation driven by the fluctuating ambient noise levels detected by the processor. Together, the presence of behavioral and NRT responses evidences successful electric stimulation of the auditory system.

There are several unique technical challenges to cochlear implant design and surgery in the mouse compared to other species. Scala tympani length and volume are less than that of other mammalian CI model species, which requires a smaller diameter and length electrode array[48]. The CI presented here was designed specifically for mouse implantation, with an intracochlear diameter of 0.15mm and insertion length of 2.25mm, which would approximate the 23.17kHz frequency region[54] if perfectly following the organ of Corti. The relatively small size of the mouse body and skull prevents placement of the entire implant and transcutaneous connector within the head, necessitating routing of the lead wire and connector across the neck to the dorsum. This configuration introduces a point of repeated strain with head rotation and flexion / extension, predisposing the lead wire to fracture. Helixed wire was implemented to provide strain resistance, which reduced, but did not eliminate the occurrence of lead wire fractures. Additionally, the extracochlear portions of the CI included a thicker silicone insulation for further strain resistance. The potential translation of extracochlear lead wire strain to the intracochlear portions was mitigated by stable fixation of the implant within the tympanic bulla with dental cement.

The custom stimulation cage allowed continuous connectivity of the CI within the normal mouse housing environment, allowing repeated electric stimulation sessions and impedance and NRT recordings. This system improves ease of experimentation and may reduce animal stress associated with handling, change in housing environment and repeated anesthetic exposure [55]. Inclusion of the soft harness is necessary to reduce strain on the tether and transcutaneous connector of the CI as the mouse moves freely around the cage, facilitated by the swiveling commutator.

Hearing preservation cochlear implantation has significantly expanded the CI candidate population and has shown the added benefit of combined electric and acoustic hearing [1–6, 8]. However, the mechanism for delayed loss of residual acoustic hearing affecting a subset of patients is still not fully elucidated. This study demonstrated the ability for hearing preservation cochlear implantation in normal hearing mice, with stable low frequency (8 and 16kHz) hearing outcomes over 6 weeks. Hearing loss was seen at the higher 32kHz frequency tested, which may be secondary implant insertion past the corresponding cochlear frequency region. Hearing levels were stable over the entire course of the experiment, without any delayed loss of residual post-implantation hearing. Although likely multifactorial, delayed loss of residual acoustic hearing may be influenced by electric stimulation characteristics, the pre-operative hearing loss etiology, and perhaps gender [11]. Thus, future studies of delayed loss of residual acoustic hearing after hearing preservation cochlear implantation should consider the factors of pre-operative partial deafening, electric stimulation and sex of the animals. Notably, hearing assessments were deferred from the main chronic stimulation study to reduce mortality risk from repeated exposure to ketamine and xylazine needed for serial ABR and DPOAE testing. Although isoflurane inhaled anesthesia alters ABR and DPOAE responses [56], it may be incorporated into other elements of the experimental protocol to reduce total injectable anesthesia exposure and related mortality.

A robust peri-implant tissue response confined to the scala tympani was seen in all subjects to a varying degree, composed of both soft tissue and neo-ossification. A similar tissue response has been seen 7 days after passive cochlear implantation of nylon monofilament in Balb/c mice [39] and following placement of silicone implants that mimic the insulation of CI electrode arrays[46]. However, the time course to develop a similar peri-implant tissue response may be longer in the guinea pig [22, 36] and cat [26]. The soft tissue response commonly extended from the round window to an area just distal to the electrode tip. Neo-ossification presented in a varied, non-continuous pattern, frequently involving the round window with isolated foci distally. This physical variance highlights the utility of the quantitative 3D volumetric segmentation approach to avoid sampling errors that may occur when quantifying only selected histologic sections or image slices. Human temporal bone data have shown the pattern of current flow in full versus half banded cochlear implants may affect the tissue response [16]. The current study did not find any significant difference in the fractional volume of soft tissue or neo-ossification between stimulated and non-stimulated subjects. However, this analysis is limited by sample numbers and varying duration of implantation among subjects and the study was not designed to specifically investigate such an effect.

The dual approach of non-destructive 3D x-ray microscopy followed by classic histologic sectioning shows complimentary utility. 3D x-ray microscopy provided reliable anatomic cochlear detail with delineation of the tissue response elements, facilitating easier quantification via 3D volume segmentation. The mean scala tympani volume measured in this study is similar to that found in other microCT studies [48, 57], adding confidence to the 3D volume segmentation strategy used. Further, the ability to co-register images of the implant within the cochlea may enable more specific spatial analysis relative to electrode orientation. The follow-up histologic preparation provides greater cellular detail of neurosensory and inflammatory elements than 3D x-ray microscopy.

The face validity [58] of this model is supported by several experimental design elements and biologic similarities between humans and mice. The mouse CI is fabricated with silicone and platinum materials similar to those used in human implants. CI material composition may play an important role in the peri-implant tissue response through a foreign body reaction [20, 21]. Extending design similarities, human CI processors, programming strategies and clinical software are used for both stimulation and objective measurements. The durability of the implant and susceptibility to failure by microfracture does limit the model, however design strategies have been implemented to mitigate these factors. Further, x-ray imaging, behavioral responses and NRT recordings can confirm correct placement and function of the implant so that device failure does not confound results. The overall structure and function of the human and mouse cochlea is similar, but with differences in overall size, number of cochlear turns and frequency range of hearing. Our study reproduced the intracochlear tissue response seen in human temporal bones [14, 18–21], adding to overall model validity. However, susceptibility to cochlear insults differs among mouse strains and this finding may differ in other non-CBA/J mice. The current study was limited by the lack of pre-operative hearing loss in implanted subjects. Several valid human disease simulations of presbycusic, hereditary, ototoxic and noise-induced hearing loss exist in the mouse [40, 41]. Additionally, it is not known whether sex plays a role in tissue responses and hearing loss following cochlear implantation, although one study identified male sex as a risk factor for hearing loss following CI in humans [10]. As the goal of this work was to develop a reliable, useful mouse CI model, our study was not designed or powered to detect sex differences among the various outcome measures. Future studies may combine this model of cochlear implantation with established hearing loss disease simulations, in both genders, to create a valid disease model of traditional and hearing preservation cochlear implantation that can be probed with the vast genetic and molecular toolkit uniquely available to the mouse.

## Conclusions

We describe a mouse model of cochlear implantation with chronic electric stimulation and the potential for hearing preservation implantation. The intracochlear tissue response seen in some human temporal bones is robustly reproduced in the CBA/J mouse, which may allow future mechanistic and therapeutic studies. The molecular and genetic research techniques available to the mouse, in addition to the radiologic, histologic and objective measures methods described here, provides unique advantages over other model species to CI biology investigations. Future studies with this model should employ established mouse models of hearing loss to more closely mirror human cochlear implantation recipients.
